# Lead‐Dominated Hyperfine Interaction Impacting the Carrier Spin Dynamics in Halide Perovskites

**DOI:** 10.1002/adma.202105263

**Published:** 2021-10-24

**Authors:** Erik Kirstein, Dmitri R. Yakovlev, Mikhail M. Glazov, Eiko Evers, Evgeny A. Zhukov, Vasilii V. Belykh, Nataliia E. Kopteva, Dennis Kudlacik, Olga Nazarenko, Dmitry N. Dirin, Maksym V. Kovalenko, Manfred Bayer

**Affiliations:** ^1^ Experimentelle Physik 2 Technische Universität Dortmund 44227 Dortmund Germany; ^2^ Ioffe Institute Russian Academy of Sciences St. Petersburg 194021 Russia; ^3^ P. N. Lebedev Physical Institute of the Russian Academy of Sciences Moscow 119991 Russia; ^4^ Laboratory of Inorganic Chemistry Department of Chemistry and Applied Biosciences ETH Zürich Zürich CH‐8093 Switzerland; ^5^ Laboratory for Thin Films and Photovoltaics Empa‐Swiss Federal Laboratories for Materials Science and Technology Dübendorf CH‐8600 Switzerland

**Keywords:** ABX_3_, carrier spin coherence, dynamic nuclear polarization, hyperfine interaction, perovskites, spintronics

## Abstract

The outstanding optical quality of lead halide perovskites inspires studies of their potential for the optical control of carrier spins as pursued in other materials. Entering largely uncharted territory, time‐resolved pump–probe Kerr rotation is used to explore the coherent spin dynamics of electrons and holes in bulk formamidinium caesium lead iodine bromide (FA_0.9_Cs_0.1_PbI_2.8_Br_0.2_) and to determine key parameters characterizing interactions of their spins, such as the *g*‐factors and relaxation times. The demonstrated long spin dynamics and narrow *g*‐factor distribution prove the perovskites as promising competitors for conventional semiconductors in spintronics. The dynamic nuclear polarization via spin‐oriented holes is realized and the identification of the lead (^207^Pb) isotope in optically detected nuclear magnetic resonance proves that the hole–nuclei interaction is dominated by the lead ions. A detailed theoretical analysis accounting for the specifics of the lead halide perovskite materials allows the evaluation of the underlying hyperfine interaction constants, both for electrons and holes. Recombination and spin dynamics evidence that at low temperatures, photogenerated electrons and holes are localized at different regions of the perovskite crystal, resulting in their long lifetimes up to 44 μs. The findings form the base for the tailored development of spin‐optoelectronic applications for the large family of lead halide perovskites and their nanostructures.

## Graphical abstract

Building up on the excellent optical properties of perovskite materials, the electron and hole spin dynamics provide fundamental insights into the carrier–nuclear spin system. In the model perovskite, bulk FA_0.9_Cs_0.1_PbI_2.8_Br_0.2_, Landé factors, key spin relaxation times, and mechanisms are determined. Optically detected NMR reveals the dominance of the lead spin hyperfine interaction at the top of the valence band.

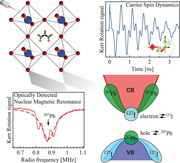

## 1 Introduction

Synthesis, characterization, and investigation of perovskites, mostly of the hybrid organic–inorganic lead halide perovskites, for example, MAPbI_3_ and FAPbI_3_, continue to evolve rapidly. The interest has been boosted by photovoltaic applications, as their quantum efficiency has reached 25.5%^[^ ^1^ ^]^ but also extending to radiation‐sensing^[^ ^2^, ^3^ ^]^ and a variety of optoelectronic devices.^[^ ^4^, ^5^, ^6^, ^7^ ^]^ Reaching the limits of high‐quality MAPbI_3_, FAPbI_3_, and CsPbI_3_ single crystals, combined structures with MA, FA, and caesium (Cs) cation mixture became the state of the art perovskite materials, increasing quantum efficiency and prolonging structural stability from days to months.^[^ ^2^, ^8^, ^9^, ^10^ ^]^ Still the fundamental physical properties are close to their parent structures, thus the presented FA_0.9_Cs_0.1_PbI_2.8_Br_0.2_ acts as a valid model system for the class of lead halide perovskites.

Compared to conventional III‐V and II‐VI semiconductors, the perovskites have in some sense an inverted band structure: the valence band (VB) states are formed by *s*‐orbitals, while the conduction band (CB) states are contributed by *p*‐orbitals. The strong spin–orbit coupling and in particular the Rashba effect,^[^ ^11^, ^12^, ^13^, ^14^ ^]^ also exchanges the spin properties of electrons and holes.^[^ ^15^, ^16^ ^]^ As a consequence, the hyperfine interaction with the lattice nuclei is dominated by the holes and not by the electrons.

The perovskite band structure gives clean polarization selection rules for the optical transitions so that in combination with the excellent optical properties it should be rewarding to seek for methods of optical manipulation of the carrier spins. Indeed, there has been already a number of proof‐of‐principle studies corroborating that a similar level of optical spin control may be reached as in other material classes. In particular, established techniques for studying spin‐dependent phenomena could be transferred to the perovskites: optical orientation^[^ ^6^, ^17^, ^18^, ^19^, ^20^ ^]^ and optical alignment,^[^ ^18^ ^]^ polarization‐resolved emission in magnetic field,^[^ ^21^, ^22^, ^23^ ^]^ pump–probe time‐resolved Kerr rotation (TRKR),^[^ ^24^, ^25^ ^]^ and nuclear magnetic resonance (NMR).^[^ ^26^, ^27^, ^28^ ^]^ The perovskite materials seem to be highly interesting for spin‐based information technologies, also in the quantum regime.^[^ ^29^ ^]^ Beyond the mentioned works, however, reports on the spin physics in perovskites are scarce and also provide controversial conclusions. This calls for a systematic investigation of the perovskite spin properties, which can provide further exclusive insight into their electronic properties.^[^ ^30^, ^31^, ^32^ ^]^

Here, we apply pump–probe Kerr rotation implemented in Faraday, Voigt, and tilted magnetic field configurations with circular and alternating circular polarized excitation^[^ ^33^ ^]^ to study the coherent spin dynamics in FA_0.9_Cs_0.1_PbI_2.8_Br_0.2_ perovskite single crystals at cryogenic temperatures, applying also strong magnetic fields. We extend the range of the methods applied previously to address the spin dynamics of perovskites and access the nuclear spin dynamics and coupling of the charge carriers and nuclear spins by means of the optically induced dynamical nuclear polarization and optically detected nuclear magnetic resonance (ODNMR); see the Experimental Section. Thereby we develop a comprehensive picture of the parameters that characterize the spin systems. These include the spin coherence and relaxation times *T*_1_ and $T_{2}^{*}$, the electron and hole *g*‐factors, and the hyperfine interaction constants of carriers with the nuclear spins that are extracted from dynamic nuclear polarization (DNP). Using ODNMR we identify the dominating role of the ^207^Pb isotope in the hyperfine interaction and address the mechanisms of the hyperfine coupling in perovskites based on the theory–experiment comparison.

Optical spin control allows for ultrafast manipulation on timescales shorter than energy loss via spin relaxation, so that spin coherence is maintained providing potentially high preservation energy efficiency. Relating the measured spin parameters to the material properties gives insight on how the perovskite materials could be tailored to optimize the spin properties for the envisioned applications.

## 2 Results

### 2.1 Exciton Recombination and Initial Spin Dynamics

**Figure 1:**
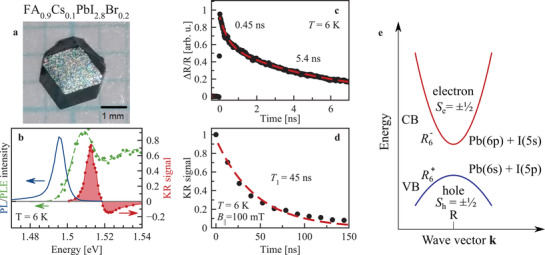
Exciton recombination and spin relaxation dynamics in FA_0.9_Cs_0.1_PbI_2.8_Br_0.2_. a) Microscopy image of FA_0.9_Cs_0.1_PbI_2.8_Br_0.2_ crystal. b) Spectra of photoluminescence (blue) excited at 1.96 eV and photoluminescence excitation (green), as well as of TRKR amplitude at zero time delay (red). c) Differential reflectivity dynamics Δ*R*/*R* (dots) and fitted with bi‐exponential function (line). d) Dynamics of KR amplitude (dots) with mono‐exponential fit (line) at *B*_||_ = 100 mT. The dynamics in (c,d) are measured with the laser photon energy of 1.513 eV. e) Sketch of direct bandgap at the R point of the Brillouin zone. Valence band is formed by 6*s*‐type lead $|s_{\text{Pb}}\rangle$ orbitals strongly hybridized with iodine 5*p*‐type $|p_{I}\rangle$ orbitals, conduction band is formed by 6*p*‐type lead orbitals with minor admixture of 5*s* iodine orbitals. Nomenclature follows ref. [41] and *S*_e,h_ here denotes the component of the angular momentum of the Bloch function.

FA_0.9_Cs_0.1_PbI_2.8_Br_0.2_ perovskite crystals have a direct bandgap at the R‐point (the cube corner along the [111]‐direction) of the Brillouin zone. The band structure is sketched in **Figure** **1**e. The valence band (VB) is formed by Pb(6*s*)−I(5*p*) antibonding orbitals, while the conduction band (CB) is mainly contributed by Pb(6*p*) orbitals, with only weak mixing with iodine 5*s* orbitals.^[^ ^34^, ^35^, ^36^, ^37^ ^]^ The VB Pb–I mixing ratio is estimated to be close to 1:3, while the CB has only about 5% I(5*s*) contribution.^[^ ^38^ ^]^

The low‐temperature photoluminescence (PL) in Figure 1b with a maximum at 1.495 eV arises likely from bound excitons. In photoluminescence excitation (PLE) the free exciton absorption has its maximum at 1.508 eV. The spectrum of the TRKR signal (red area) has its maximum at 1.512 eV, coinciding basically with the exciton resonance. The asymmetric lineshape is typical for resonant KR signals.^[^ ^39^ ^]^ In all following experiments the laser is tuned to the KR maximum.

The comparison of the decay of the differential reflectivity Δ*R*/*R* (probing the exciton population) and KR (probing the induced spin polarization), Figures 1c and 1d, respectively, show that their dynamics are strongly different. The population dynamics show a bi‐exponential decay with times of 0.45 and 5.4 ns. In contrast, the spin dynamics measured by the extended pump–probe KR technique^[^ ^40^ ^]^ in *B*_||_ = 100 mT show a mono‐exponential decay with the longitudinal spin relaxation time *T*_1_ = 45 ns. It exceeds by an order of magnitude the population times indicating that resident carriers control the KR dynamics.^[^ ^25^ ^]^

### 2.2 Coherent Spin Dynamics of Electrons and Holes

**Figure 2:**
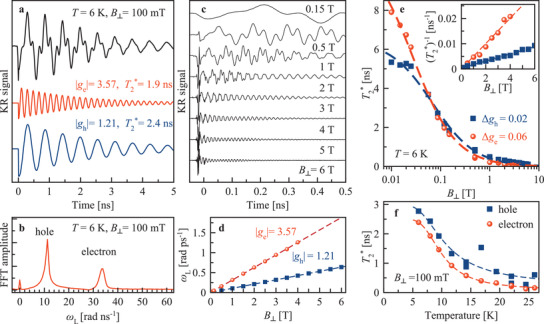
Spin dynamics of resident carriers in Voigt geometry. a) Time‐resolved Kerr rotation signal at *B*_⊥_ = 100 mT (black), contributed by electron (red) and hole (blue) oscillating components shown individually below. b) Fast Fourier transformation (FFT) spectrum of the KR signal in (a). c) TRKR for various magnetic field strengths. Signals are shifted vertically for clarity and are not normalized. With increasing field, the Larmor frequency grows and $T_{2}^{*}$ shortens. d) Larmor frequency dependence on magnetic field extracted by fit from (c) (symbols). Linear fits (lines) give |*g*_e_| = 3.57 and |*g*_h_| = 1.21. e) $T_{2}^{*}$ dependence on magnetic field for electrons and holes. Note the logarithmic scale for the magnetic field. Lines are fits along Equation (1) with Δ*g*_e_ = 0.06 and Δ*g*_h_ = 0.02. In the inset, data and fits are rescaled to show ${\left(T_{2}^{*}\right)}^{-1}$ in linear scale. f) Temperature dependence of $T_{2}^{*}$ (symbols). The corresponding TRKR series are presented in Figure S3a, Supporting Information. Lines are Arrhenius fits with *E*_A,e_ = 3.8 meV for electrons and *E*_A,h_ = 2.9 meV for holes. Data in panels (a–e) are measured at *T* = 6 K.

By applying a magnetic field perpendicular to the light propagation direction (Voigt geometry, *B*_⊥_), along which the carrier spins are oriented by pulsed optical excitation, spin quantum beats are launched, giving access to the carrier *g*‐factors and spin relaxation times. In **Figure** **2**a the TRKR signal is shown at *B*_⊥_=100 mT. The seemingly irregular beats result from superposition of two oscillating components which can be distinguished in the fast Fourier transform spectrum (Figure 2b). As shown below, one component originates from the high frequency electron spin precession and another one from the slower hole spin precession. With increasing magnetic field the dynamics is accelerated: the oscillation frequencies and decay rates increase (Figure 2c). From the linear dependencies of the Larmor frequencies on magnetic field (Figure 2d) the *g*‐factor moduli of electrons (|*g*_e_| = 3.57) and holes (|*g*_h_| = 1.21) are determined. The identification of the carrier type is facilitated by the dependence of the *g*‐factors in the perovskites on the bandgap energy: For the relevant *E*_g_ ≈ 1.5 eV, k · p theory and atomistic approaches give *g*_e_ > 0, *g*_h_ < 0, and |*g*_e_| > |*g*_h_|.^[^ ^15^, ^38^ ^]^ There are several factors assuring that the KR signals arise from resident electrons and holes that are weakly localized in different crystal sites, similar to CsPbBr_3_ crystals.^[^ ^25^ ^]^ Among them are the long dephasing times $T_{2}^{*}$ of the KR signals exceeding the exciton recombination time, the strong temperature dependence of $T_{2}^{*}$ (Figure 2f), and the linear dependence of the Larmor frequency on the magnetic field down to the lowest applied fields (Figure 2d), confirming the absence of electron–hole exchange effects and evidencing that the electrons and holes are not bound to excitons.

We measured the *g*‐factors by varying the magnetic field direction using a vector magnet. The *g*‐factor anisotropy is small in FA_0.9_Cs_0.1_PbI_2.8_Br_0.2_, not exceeding 3% for electrons and 4% for holes (Figure S5, Supporting Information), which is in line with the close‐to‐cubic crystal structure. The decay of the KR signal envelopes in Figure 2a gives the spin dephasing time $T_{2}^{*}$. At *B*_⊥_ = 100 mT it is $T_{2,e}^{*}=1.9$ ns for the resident electrons and $T_{2,h}^{*}=2.4$ ns for the resident holes. $T_{2}^{*}$ shortens with increasing magnetic field (Figure 2e), as expected for spin ensembles with a spread of *g*‐factors Δ*g*.^[^ ^33^ ^]^ Fitting the $T_{2}^{*}$ data with

(1) $$1/T_{2}^{*}(B)=1/T_{2,0}^{*}+\Delta g\mu_{B}B/\hbar$$

we get Δ*g*_e_ = 0.06 and Δ*g*_h_ = 0.02 for the electrons and holes, respectively. The electron spin dephasing time extrapolated to zero magnetic field $T_{2,0}^{*}=8$ ns is longer than the hole one of 5.5 ns.

Interestingly, the spin dephasing accelerates with increasing temperature, see Figure 2f. We describe the temperature dependence of $T_{2}^{*}$ with an Arrhenius‐like function $1/T_{2}^{*}(T)=(1/T_{2,0}^{*})+w\exp(-E_{A}/k_{B}T)$. The constant *w* characterizes the strength of the carrier–phonon interaction (0.03 ps^−1^ for electrons and 0.06 ps^−1^ for holes), *E*_A_ are the activation energies (*E*_A,e_ = 3.8 meV and *E*_A,h_ = 2.9 meV), and *k*_B_ is the Boltzmann constant. It is evident from the activation energies that the resident carriers are localized by shallow potential fluctuations. The carrier localization allows pronounced DNP and thus gives access to the coupled carrier–nuclei spin dynamics.

### 2.3 Dynamic Nuclear Polarization

**Figure 3:**
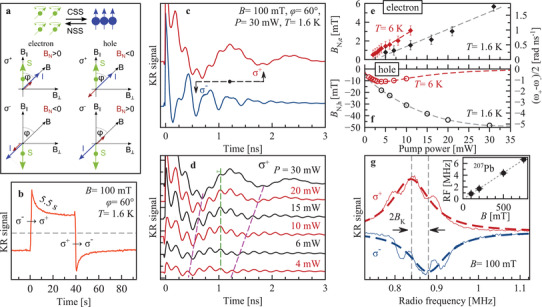
Dynamic nuclear polarization and ODNMR. a) Top, scheme of interaction of carrier spin system (CSS) and nuclear spin system (NSS). The CSS polarizes the NSS and the NSS in turn causes an Overhauser field acting back on the CSS.^[^ ^44^ ^]^ The carrier spin direction *S* is determined by the light helicity, σ^+^ (σ^−^) corresponds to spin up (down). The nuclear polarization *I* (blue arrow) builds up in the direction of *S* (green arrow) along the magnetic field *B* (black arrow). Here the magnetic field is inclined relative to the carrier spin polarization by the angle φ. For a σ^+^ polarized pump, the nuclear spin polarization manifests as Overhauser field *B*_N_ (violet arrow), which for electrons (*g*_e_ > 0) is directed along *I* and for holes (*g*_h_ < 0) in the opposite direction. The CSS produces the Knight field *B*_K_ acting on the NSS, not sketched. b) Dynamics of DNP. Sequence of polarization: 2 min σ^−^, 40 s σ^+^, 2 min σ^−^. Repolarization of nuclei occurs with a time of 5.5 s. Time delay between pump and probe pulses is 500 ps, pump power is 20 mW. c) TRKR with σ^+^ (red) and σ^−^ (blue) pump. The Overhauser field is either parallel or antiparallel to the external magnetic field. The arrows indicate the time shift of the second minimum of the hole spin Larmor precession. d) TRKR measured at different pump powers. The change of ω_L_ is traced for the first and second minima with purple dashed lines for hole precession, and for the fifth maximum for the electron with the green line. *T* = 1.6 K. e and ,f) Dependence of *B*_N, e(h)_ on pump power at *T* = 1.6 K (black) and 6 K (red). Right scale shows the change of Larmor frequency. g) ODNMR resonance of ^207^Pb for σ^+^ and σ^−^ pump measured at time delay of 960 ps and pump power of 20 mW. *T* = 1.6 K. The inset shows linear dependence of the resonance frequency on magnetic field with a slope of 8.80 MHz T^−1^. Note, results with the RF field are given in (g) only. For all experiments in this figure *B* = 100 mT and φ = 60°.

To study the hyperfine interaction of carrier spins with the nuclear spin bath we induce a DNP: the spin polarization of carriers optically oriented by a pump beam of fixed helicity is transferred by flip‐flop processes to the nuclear spin system (NSS), which consequently becomes polarized. The nuclear spin polarization 〈**I**〉 is given by^[^ ^42^, ^43^ ^]^

(2) $$\left\langle I\right\rangle =l\frac{4I(I+1)}{3}\frac{B(B\cdot \left\langle S_{e(h)}\right\rangle )}{B^{2}}$$

with **I** being the nuclear spin, **S**_e(h)_ the steady‐state polarization of carriers induced by the optical orientation, and *l* the leakage factor characterizing the losses of nuclear polarization due relaxation processes other than the hyperfine coupling. Note that a DNP develops only if **S**_e(h)_ and **B** are not perpendicular to each other, as otherwise **B**·**S**_e(h)_ = 0. A detailed analysis of the DNP mechanisms is presented in Section S2.4, Supporting Information. The induced nuclear polarization **I** is collinear to **B**. The polarized nuclear spins act back on the carriers by means of the Overhauser field **I**_N_, which is proportional to **I** and collinear to it and, thus, to **B**. The Overhauser field adds to the external magnetic field, changing the frequency of the carrier spin precession in the total field *B* ± *B*_N,e(h)_, as ω_L,e(h)_ = |*g*_e(h)_|μ_B_(*B* ± *B*_N,e(h)_)/*ℏ*. The magnitude of this change is a measure of the nuclear spin polarization or $B_{N’\text{(eh)}}$. The direction of $B_{N}{,}_{e(h)}\;=A_{e(h)}I/(g_{e(h)}\mu_{B})$ is determined by the sign of the hyperfine coupling constant *A*_e(h)_, the carrier *g*‐factor, and the direction of **I**. The last one is governed by $S_{e(h)}$, see Equation (2), and can be adjusted by varying the pump helicity. The DNP process is shown schematically in **Figure** **3**a.

For the experimental demonstration we choose *B* = 100 mT with an angle between the field and the photoinduced carrier spin polarization (i.e., the pump wave vector direction) of φ = 60°. Figure 3c shows a remarkable difference in the Kerr rotation dynamics when we change the pump‐light helicity between σ^+^ and σ^−^. The strongest effect occurs for the hole Larmor precession frequency, which changes from ω_L,h_ = 16 rad ns^−1^ for σ^−^ pumping down to 5 rad ns^−1^ for σ^+^ pumping, see the second minimum position marked by the arrows in Figure 3c. Note, that under conditions when DNP is absent (modulated pump helicity in the Voigt geometry) at *B*_⊥_ = 100 mT, ω_L,h_ = 11 rad ns^−1^ (Figure 2b). The DNP effect, although smaller, is also present for the electrons whose Larmor frequency changes between 28 and 32 rad ns^−1^ for σ^−^ and σ^+^ pump, respectively, while without DNP ω_L,e_ = 30 rad ns^−1^.

The DNP‐induced changes of the Larmor frequency for electrons and holes are opposite: for σ^−^ pump the hole frequency increases, but the electron one decreases. This means that for σ^−^ excitation, **B**_N,h_ is parallel to the external magnetic field, while **B**_N,e_ is antiparallel to **B**. This is in line with our theoretical analysis, which shows that: i) the direction of **I** is the same for the DNP via electrons and holes, ii) *g*_e_ > 0 and *g*_h_ < 0, and iii) the hyperfine coupling constants *A*_e_ and *A*_h_ are positive.

The DNP‐induced nuclear field is determined by the spin flux from the optically oriented carriers to the NSS and, therefore, depends on the pump power. One can see in Figure 3d the prominent decrease of the hole Larmor frequency, reflecting increase of DNP, when the power of the σ^+^ pump increases from 4 up to 30 mW. The corresponding changes of the Overhauser fields are given in Figure 3e,f. With increasing pump power, the absolute values of the Overhauser fields increase monotonically, up to *B*_N,h_ ≈ −50 mT for holes and *B*_N,e_ ≈ 6 mT for electrons. The increase scales linearly with power, until indications for saturation of *B*_N,h_ are found at *P* > 20 mW, while for the electrons saturation is absent. Also, at elevated temperature of 6 K the pump power dependencies of the Overhauser fields acting on electron and hole differ: The temperature effect on *B*_N,h_ is stronger resulting in a non‐monotonic power dependence of the hole Overhauser field, which can be related to a variation of the leakage factor due to the temperature and pump power dependence of the carrier‐induced flip‐flop processes, see Supporting Information for details.

The DNP dynamics was assessed through the time‐resolved changes of the KR amplitude at 0.5 ns pump–probe delay after abrupt switching between the σ^+^ and σ^−^ pump helicities. One can see in Figure 3b that the repolarization time is 5.5 s, which is in the same range of times reported for III‐V and II‐VI semiconductors.^[^ ^45^, ^46^ ^]^

### 2.4 Optically Detected Nuclear Magnetic Resonance

In order to identify the nuclear isotopes dominating the hyperfine interaction with electrons and holes we apply ODNMR. To prepare the system, the nuclear spins are polarized via DNP by pumping with fixed helicity at *B* = 100 mT in tilted geometry (φ = 60°). The carrier spins precess in the combined external and Overhauser field. Subsequently, a variable frequency RF field is applied along the optical axis. When the RF matches the nuclear spin splitting of an isotope, the nuclear spins are depolarized reducing the Overhauser field. Consequently, the Larmor frequency of the carrier spins changes which leads to a change of the KR amplitude at a given pump–probe delay chosen to be 960 ps. We found only one prominent NMR resonance in the RF range of 100 Hz to 10 MHz, shown in Figure 3g. The resonance maximum occurs at 0.840 MHz for σ^+^ and at 0.880 MHz for σ^−^ pump helicity. The mean value is 0.860 MHz and the difference of 20 kHz originates from polarized carrier spins, namely holes as shown below, which act as an additional effective field *B*_K_ = 2 mT on the nuclear spins (Knight field). The magnetic field dependence of the NMR frequency corrected for *B*_K_ is plotted in the inset of Figure 3g. It has a slope of 8.80 MHz T^−1^. In FA_0.9_Cs_0.1_PbI_2.8_Br_0.2_ two nuclear isotopes have a vacuum gyromagnetic ratio close to this value ^207^Pb with γ = 8.882 MHz T^−1^ (natural abundance of 22.1%, *I*_Pb_ = 1/2) and ^127^I with γ = 8.578 MHz T^−1^ (natural abundance of 100%, *I*_I_ = 5/2),^[^ ^47^ ^]^ see Table S1, Supporting Information. A wide range of chemical shifts has been reported for ^207^Pb, for instance in the case of metallic Pb −11 kppm,^[^ ^48^ ^]^ which corresponds to a gyromagnetic value of 8.784 MHz T^−1^. Together with the theoretical analysis we conclude that it is the ^207^Pb isotope that is observed in ODNMR. The resonance full width at half maximum (FWHM) is Γ_N_ = 100 kHz, which corresponds to a nuclear spin dephasing time of $T_{2,\text{Pb}}^{*}=1/(\pi \Gamma_{N})\approx 3\;\mu s$.^[^ ^49^ ^]^ Our ODNMR experiments show practically no effect on the Overhauser field experienced by the electron, see Supporting Information for further experimental details. Thus, it is unlikely that the dynamic polarization of the ^207^Pb nuclei produces an Overhauser field experienced by the electrons. A natural candidate for the hyperfine interaction with the conduction band electrons is ^127^I. As compared to ^207^Pb, its spin is large and quadrupole coupling can broaden its NMR. Also, the Overhauser field acting on the electrons is weaker as compared to that acting on the holes, in agreement with small fraction of the I orbitals in the conduction band wavefunction.

### 2.5 Discussion

The measured electron and hole Landé factors in FA_0.9_Cs_0.1_PbI_2.8_Br_0.2_ perovskite crystals are *g*_e_ = 3.57 and *g*_h_ = −1.21, the largest reported so far for lead halide perovskites. These values are in line with model predictions^[^ ^15^ ^]^ and are comparable with the *g*_e_ = 2.63 and *g*_h_ = (−)0.33 reported for MAPbCl_*x*_I_3−*x*_ (with small *x*) with a somewhat larger bandgap energy of 1.64 eV.^[^ ^24^ ^]^ The spin precession is characterized by long spin dephasing times: $T_{2,0,e}^{*}=8\;\text{ns}$ and $T_{2,0,h}^{*}=5.5\;\text{ns}$. They are considerably longer than those reported for MAPbCl_*x*_I_3−*x*_ polycrystalline films (<1 ns),^[^ ^24^ ^]^ and for CsPbBr_3_ crystals with $T_{2,0,e}^{*}=5.2\;\text{ns}$ and $T_{2,0,h}^{*}=1.9\;\text{ns}$.^[^ ^25^ ^]^ The spin dephasing accelerates with increasing magnetic field and shows an activation behavior with rising temperature. Such a behavior is characteristic for localized carriers, which spin relaxation at zero field is controlled by the hyperfine interaction with the host lattice nuclei and at non‐zero field by the *g*‐factor dispersion. The spin dephasing times allow us to estimate the number of the nuclei spins interacting with the resident holes and electrons, and also their localization size (see Section S2.5, Supporting Information). The resident hole interacts with about 10^4^ nuclei of ^207^Pb, and the resident electron with about 7 × 10^4^ nuclei of ^127^I. Localization length for both charge carriers is about 10 nm. We emphasize that the resident electrons and holes are localized at different locations, which seems to be a specific feature of lead halide perovskite materials. It is also confirmed by non‐exponential decay of the photoluminescence with the longest time reaching 44 μs, see Section S3.5, Supporting Information.

The longitudinal spin relaxation time *T*_1_ = 45 ns is significantly longer than $T_{2}^{*}$, and comparable to *T*_1_ = 53 ns in CsPbBr_3_ crystals.^[^ ^25^ ^]^ We cannot unambiguously ascribe it to electrons or holes, therefore, this value can be considered as the shortest time among them.

The measured spin dephasing and spin relaxation times of carriers exceed the exciton lifetime as confirmed by the time‐resolved differential reflectivity. This implies that either the photogenerated charge carriers separate in space before recombination, or the optically induced spin polarization of excitons is transferred to the resident carriers.^[^ ^25^ ^]^ While further experiments are needed to pinpoint the relevant scenario, our results clearly demonstrate long‐living spin coherence of electrons and holes. Similar to conventional III‐V and II‐VI semiconductors,^[^ ^32^ ^]^ the long‐living carrier spin dynamics in FA_0.9_Cs_0.1_PbI_2.8_Br_0.2_ perovskite crystals is controlled by the hyperfine interaction with the lattice nuclei, as follows from estimates of the dephasing and relaxation times, cf. ref. ^[^ ^25^ ^]^. and Supporting Information, and is directly confirmed by the measured DNP.

Our analysis shows that the ^207^Pb isotope dominates the hyperfine interaction with the hole spins, whereas we observe basically no effect of any isotope spin on the Overhauser field experienced by the electron spins. Indeed, the top of the VB is mainly formed by *s*‐type Pb orbitals with significant contact interaction (see ref. ^[^ ^25^ ^]^ and Supporting Information). This conjecture is confirmed by the observation of the ODNMR of ^207^Pb via the Knight field of 2 mT generated by the hole spins (7% hole spin polarization, see Section S2.5, Supporting Information for details), making it possible to also study the nuclear polarization dynamics and extract the nuclear relaxation time *T*_1N_ ≈ 5.5 s in our conditions. A nuclear spin relaxation time in the range of seconds is characteristic for semiconductors,^[^ ^32^ ^]^ which confirms and highlights that the perovskites offer bright spin physics on top of excellent optical properties.

## 3 Experimental Section

### Samples

Here, α‐phase FA_0.9_Cs_0.1_PbI_2.8_Br_0.2_ (FA is formamidinium) solution‐grown single crystals were studied. The α‐phase (black phase) exhibited cubic crystal structure^[^ ^50^ ^]^ and was oriented such that the [001] direction points along the light wave vector **k**. For FA_0.9_Cs_0.1_PbI_2.8_Br_0.2_, the Goldschmidt tolerance factor *t* was close to 1, thus the orthorhombic distortion was small and FA_0.9_Cs_0.1_PbI_2.8_Br_0.2_ can be assumed to be cubic in the first instance. The pseudo‐cubic lattice constant for hybrid organic perovskite (HOP) was around 6.3 A, but not determined for this specific sample. For more details the reader was refereed to ref. [2] and Section S1, Supporting Information. Note that no strong indications (in temperature ‐dependent PL and *g*‐factor anisotropy) for a phase transition toward the orthorhombic phase were observed. For the optical experiments in reflection geometry the sample surface was polished.

### Optical Measurements

The samples were placed in a cryostat with temperatures variable from 1.6 up to 300 K. At *T* = 1.6 K the sample was immersed in superfluid helium, while at 4.2 to 300 K it was in cooling helium gas. Two types of cryostats were used, both were equipped with superconducting magnets. The first one was a split‐coil magnet for magnetic fields up to 8 T being applied either in Faraday or Voigt geometry. The second one was a vector magnet equipped with three orthogonal pairs of split coils to orient the magnetic field up to 3 T along any chosen direction. The magnetic fields parallel to the light wave vector **k** were denoted as **B**_||_ (Faraday geometry) and magnetic fields perpendicular to **k** as **B**_⊥_ (Voigt geometry). If not stated otherwise, the **B**_⊥_ were oriented in the horizontal plane. The angle φ was defined as the angle between **B** and **k**, where φ = 0° corresponded to **B**_||_. A sketch was given in Figure 3a and Figure S5d, Supporting Information.

### Photoluminescence and Photoluminescence Excitation

The sample was excited with a 750 nm (1.653 eV) continuous‐wave (cw) diode laser with a low power of 0.5 mW. The photoluminescence was detected by a 0.5 m monochromator with a charge‐coupled‐device (CCD) camera. The PLE was measured with a tunable titanium–sapphire (Ti:Sa) continuous wave (cw) laser while recording the PL at 1.495 eV.

### Pump–Probe Time‐Resolved Kerr Rotation

The coherent spin dynamics was measured using a degenerated pump–probe setup.^[^ ^33^ ^]^ A titanium–sapphire (Ti:Sa) laser generated 1.5 ps long pulses in the spectral range of 700−980 nm (1.265−1.771 eV) with a spectral width of about 1 nm (about 1.5 meV) and pulse repetition rate of 76 MHz (repetition period *T*_R_ = 13.2 ns). The laser beam was split into two beams, in which pulses can be delayed with respect to one another by a mechanical delay line. The laser photon energy was tuned to be in resonance with the PLE maximum, for example, at 1.513 eV at *T* = 6 K. Both pump and probe beams were modulated using photoelastic modulators. The probe beam was always linearly polarized and its amplitude was modulated at 84 kHz, while the pump beam was either helicity modulated at 50 kHz between σ^+^/σ^−^ or amplitude modulated with the circular polarization fixed at σ^+^ or σ^−^. The polarization of the reflected probe beam was analyzed using a lock‐in technique with respect of its rotation (Kerr rotation). In *B*_⊥_ ≠ 0, the Kerr rotation amplitude oscillated in time reflecting the Larmor spin precession of the carriers. When both electrons and holes contribute to the Kerr rotation signal, as was the case here, the signal can be described by a superposition of two decaying oscillatory functions

(3) $$A_{\text{KR}}=S_{e}\cos(\omega_{L,e}t)\exp(-t/T_{2,e}^{*})+S_{h}\cos(\omega_{L,h}t)\exp(-t/T_{2,h}^{*})$$

The envelopes of the signal gave the spin dephasing times $T_{2}^{*}$.

### Extended Pump–Probe Kerr Rotation

The recently developed extended pump–probe technique^[^ ^40^ ^]^ was applied to lift the delay time limitation caused by the laser repetition period *T*_R_ = 13.2 ns of the used laser system. In this method both pump and probe beams were pulse picked by an electro‐optical (pump) and an acousto‐optical modulator (probe) (EOM/AOM). The EOM pulse picking can be done in such a way that only a single laser pulse with a separation of up to an integer number *n* of repetition periods toward the picked probe pulse was transmitted. This scheme conserved the ps time resolution and allowed one to measure spin dynamics up to microsecond timescales.

### Pump–Probe Time‐Resolved Differential Reflectivity

For excitation, that is, for the pump beam, the same scheme as in TRKR was used, but with linear polarization instead of circular polarization. The pump was amplitude modulated by a photo‐elastic modulator at 100 kHz. From the probe beam a reference beam was taken by a beam splitter before hitting the sample. The signal was recorded with respect to the intensity difference of the reflected probe beam relative to the reference beam with a balanced photodetector. The probe beam was balanced to zero with the pump beam blocked. Thus when the pump beam created photoexcited carriers, some states were occupied and the absorption droped. According to the Kramers–Kronig relation the reflectivity changes gave the detected signal. The differential reflectivity was sensitive to the exciton population.

### ODNMR Measurements with TRKR Detection

A small radiofrequency (RF) coil of about 5 mm diameter and 5 turns was placed close to the sample surface, similar as in ref. [46]. The coil was mounted flat on the sample surface and the laser beam was going through the core hole of the coil, see Figure S2a, Supporting Information. The RF field direction was parallel to **k** and the RF was driven by a frequency generator in the frequency range from 100 Hz up to 10 MHz, typically with a voltage of 10 V and an effective oscillating field amplitude of about 0.1 mT. The RF was terminated by internal 50 Ω resistors, but not frequency matched to the circuit. In the low frequency range up to <5 MHz the current was nearly frequency independent, as the inductive resistance was small compared to the internal termination. For the observation of DNP it is essential to incline the magnetic field away from Voigt geometry, to have a non‐zero scalar product **B** of **S** and to apply light with constant helicity. A modulated pump helicity between σ^+^ and σ^−^ polarization is used to suppress the DNP.

## Conflict of Interest

The authors declare no conflict of interest.

## Supporting information

Supporting Information

## Data Availability

The data that support the findings of this study are available from the corresponding author upon reasonable request.
