# Supplementary material for: Lead‐Dominated Hyperfine Interaction Impacting the Carrier Spin Dynamics in Halide Perovskites
Source: Adv Mater. 2021 Oct 24;34(1):2105263. doi: 10.1002/adma.202105263 (PMC11468769; doi:10.1002/adma.202105263)
Supplement: Supplementary file 1 — Supporting Information [file ADMA-34-2105263-s001.pdf]

ADVANCED MATERIALS

Supporting Information

for *Adv. Mater.,* DOI: 10.1002/adma.202105263

Lead-Dominated Hyperfine Interaction Impacting the Carrier Spin Dynamics in Halide Perovskites

Erik Kirstein, Dmitri R. Yakovlev,* Mikhail M. Glazov, Eiko Evers, Evgeny A. Zhukov, Vasilii V. Belykh, Nataliia E. Kopteva, Dennis Kudlacik, Olga Nazarenko, Dmitry N. Dirin, Maksym V. Kovalenko, and Manfred Bayer*

Supplementary Information:

Lead-dominated hyperfine interaction impacting the carrier spin dynamics in halide perovskites

Erik Kirstein,[1] Dmitri R. Yakovlev,[1,2,3] Mikhail M. Glazov,[2] Eiko Evers,[1] Evgeny A. Zhukov,[1,2] Vasilii V. Belykh,[1,3] Nataliia E. Kopteva,[1] Dennis Kudlacik,[1] Olga Nazarenko,[4] Dmitry N. Dirin,[4] Maksym V. Kovalenko,[4,5] and Manfred Bayer[1,2]

[1]*Experimentelle Physik 2, Technische Universität Dortmund, 44221 Dortmund, Germany*
[2]*Ioffe Institute, Russian Academy of Sciences, 194021 St. Petersburg, Russia*
[3]*P. N. Lebedev Physical Institute of the Russian Academy of Sciences, 119991 Moscow, Russia*
[4]*Laboratory of Inorganic Chemistry, Department of Chemistry and Applied Biosciences, ETH Zürich, CH-8093 Zürich, Switzerland*
[5]*Laboratory for Thin Films and Photovoltaics, Empa-Swiss Federal Laboratories for Materials Science and Technology, CH-8600 Dübendorf, Switzerland*

S1 Material information

The studied perovskite crystals are based on the $FAPbI_3$ material class, which crystal structure is shown in Figure S1a [1]. FA-based perovskite exhibits low trap density (1.13×10^{10} cm^{-3}) and low dark carrier density (3.9×10^9 cm^{-3}) [2, 3]. $FAPbI_3$ is chemically and thermally more stable compared to $MAPbI_3$ due to the decomposition of the latter to gaseous hydrogen iodide and methylammonium [2]. However, pure $FAPbI_3$ suffers from structural instability originating from the large size of FA cation which cannot be accommodated by inorganic perovskite framework. This instability has been successfully resolved via partial, up to 15 %, replacement of large FA cation with smaller caesium (Cs) together with iodine (I) substitution by bromide (Br) [4, 5]. As a result, the Goldschmidt tolerance factor t [6] is tuned from 1.07 in $FAPbI_3$ closer to 1 in $FA_{0.9}Cs_{0.1}PbI_{2.8}Br_{0.2}$, where it is 0.98. The band gap of $FA_{0.9}Cs_{0.1}PbI_{2.8}Br_{0.2}$ at room temperature is 1.52 eV, slightly larger than the band gap of $FAPbI_3$ of 1.42 eV [7, 2].
For crystal synthesis the inverse temperature crystallization technique is used [2, 3]. For the growth, a solution of CsI, FAI (FA being formamidinium), PbI_2, and $PbBr_2$, with GBL γ-butyrolactone as solvent is mixed. This solution is then filtered and slowly heated to 130°C temperature, whereby the single crystals are formed in the black phase of $FA_{0.9}Cs_{0.1}PbI_{2.8}Br_{0.2}$, following the reaction

$$\mathrm{PbI_2 + PbBr_2 + FAI + CsI \overset{[GBL]}{\rightarrow} FA_{0.9}Cs_{0.1}PbI_{2.8}Br_{0.2} + R.}$$

Afterwards the crystals are separated by filtering and drying. A typical crystal used for this study is shown in Figure S1b. It has a size of about 2 mm. The crystallographic analysis suggests that one of the principal axis a, b, c is normal to the front facet, thus pointing along the optical axis. In cubic approximation a=b=c. The pseudo-cubic lattice constant for hybrid organic perovskite (HOP) is around 6.3 A [8], but not determined for this specific sample.

S2 Theory of electron and hole spin dynamics in perovskites

S2.1 Basics of the band structure

Hybrid organic-inorganic lead halide perovskites, e.g. $MAPbI_3$ and $FAPbI_3$, at high temperatures have cubic symmetry with the $Pm\bar{3}m$ (221) space group and the O_h point symmetry group [9, 10]. While at low temperatures the crystals undergo phase transitions to the tetragonal or orthorhombic phases, we disregard such modifications of the crystal structure for the purposes of our study. The cubic approximation for the studied $FA_{0.9}Cs_{0.1}PbI_{2.8}Br_{0.2}$ sample, is confirmed experimentally by the almost isotropic Zeeman splitting of the electrons and holes.
In the $Pm\bar{3}m$ space group the Brillouin zone is cubic, and the direct band gap is formed at the R point, i.e., at the corner of the cube along the [111] direction. We recall that the orbital Bloch function of the

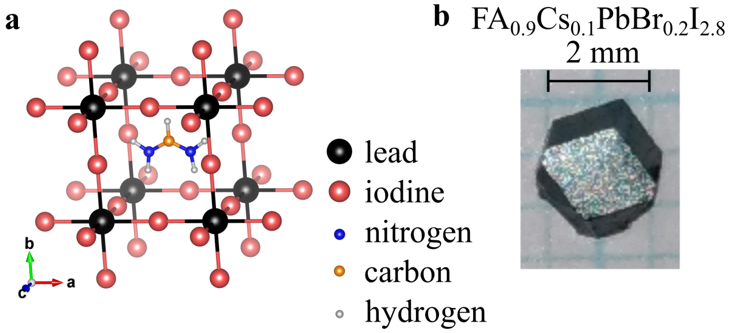

Figure S1: **a**, Simplified crystal structure of $FAPbI_3$ [1]. Black lead (Pb) atoms and red I atoms form a cubic crystal. Inside the cell is the FA molecule, with white hydrogen (H), blue nitrogen (N) and orange carbon (C) atoms. **b**, Microscopy image of $FA_{0.9}Cs_{0.1}PbI_{2.8}Br_{0.2}$ single crystal.

valence band top is invariant (R_1^+ representation [11]) and the *conduction band bottom* is formed by three orbital functions transforming according to the R_4^- representation (as vector components). With account for the spin and spin-orbit coupling (R_6^+ is the spinor representation for spin 1/2 states) one obtains for the valence band: $R_1^+ \times R_6^+ = R_6^+$, and for the conduction band: $R_4^- \times R_6^+ = R_6^- + R_8^-$. The valence band is two-fold spin degenerate. The conduction band is, in general, complex, but the bottom of the conduction band is also two-fold spin degenerate and has R_6^- symmetry.
Atomistic modeling (see, e.g., [10, 12]) demonstrates that the valence band is composed of the s-orbitals of the metal (Pb in our case), $|\mathcal{S}_0\rangle$, with an admixture of the halogen (I and Br) p-orbitals (a combination $\propto |\mathcal{X}_1\rangle + |\mathcal{Y}_2\rangle + |\mathcal{Z}_3\rangle$ with appropriate phase choice):

$$R_1^+ : \quad |v.b.\rangle = C_s^{v.b.}|\mathcal{S}_0\rangle + \frac{C_p^{v.b.}}{\sqrt{3}}\left\{|\mathcal{X}_1\rangle + |\mathcal{Y}_2\rangle + |\mathcal{Z}_3\rangle\right\}, \tag{S1a}$$

with the admixture coefficients $|C_s^{v.b.}|^2 + |C_p^{v.b.}|^2 = 1$. Here the subscript 0 refers to the Pb atom and the subscripts $1, 2, 3$ enumerate the surrounding halogens [10]. For the conduction band the main contribution comes from the p-orbitals of the metal, $|\mathcal{X}_0\rangle$, $|\mathcal{Y}_0\rangle$, $|\mathcal{Z}_0\rangle$ with an admixture of the s-orbitals of the halogen $|\mathcal{S}_1\rangle$ ($|C_p^{c.b.}|^2 + |C_s^{c.b.}|^2 = 1$):

$$R_4^- : \quad \begin{cases} |c.b., x\rangle = C_p^{c.b.}|\mathcal{X}_0\rangle + C_s^{c.b.}|\mathcal{S}_1\rangle, \\ |c.b., y\rangle = C_p^{c.b.}|\mathcal{Y}_0\rangle + C_s^{c.b.}|\mathcal{S}_2\rangle, \\ |c.b., z\rangle = C_p^{c.b.}|\mathcal{Z}_0\rangle + C_s^{c.b.}|\mathcal{S}_3\rangle. \end{cases} \tag{S1b}$$

With account for the spin-orbit coupling the Bloch amplitudes at the R-point of the Brillouin zone take the form

$$\text{valence band, } R_6^+ : \quad \begin{cases} u_{v,1/2}(\boldsymbol{r}) = |v.b.\rangle|\uparrow\rangle, \\ u_{v,-1/2}(\boldsymbol{r}) = |v.b.\rangle|\downarrow\rangle, \end{cases} \tag{S2a}$$

$$\text{conduction band: } R_6^- : \quad \begin{cases} u_{c,1/2}(\boldsymbol{r}) = -\sin\theta|c.b., z\rangle|\uparrow\rangle - \cos\theta\dfrac{|c.b., x\rangle + \mathrm{i}|c.b., y\rangle}{\sqrt{2}}|\downarrow\rangle, \\ u_{c,-1/2}(\boldsymbol{r}) = +\sin\theta|c.b., z\rangle|\downarrow\rangle - \cos\theta\dfrac{|c.b., x\rangle - \mathrm{i}|c.b., y\rangle}{\sqrt{2}}|\uparrow\rangle, \end{cases} \tag{S2b}$$

with $\uparrow$ and $\downarrow$ being the basic spinors, and $\tan 2\theta = 2\sqrt{2}$ in the cubic approximation. A sketch of the band diagram is shown in Figure 1**e** of the main text.

S2.2 Electron and hole spin dynamics

Before discussing the interplay of the carrier and nuclear spin dynamics in perovskite crystals, let us briefly analyze the mechanisms of the spin relaxation of electrons and holes unrelated to the hyperfine interac-

tion. It is well known that for free charge carriers the driving force of the spin dynamics is the spin-orbit interaction [13, 14]. Despite the fact that bulk perovskite crystals are centrosymmetric there is strong evidence for a symmetry reduction resulting in Rashba- and Dresselhaus-like contributions to the spin-orbit splitting of the conduction and valence bands [15, 16, 17]. Thus, if the electrons and holes are free to propagate in the crystal, one could expect a rather rapid spin relaxation and dephasing via the Dyakonov-Perel' mechanism (DP) with the rate [18]

$$\frac{1}{\tau_s^{(DP)}} \sim \langle \Omega_{\mathbf{k}}^2 \tau \rangle, \tag{S3}$$

where $\mathbf{\Omega_k}$ is the effective frequency of the carrier spin precession in the wavevector $\mathbf{k}$ dependent spin-orbit field, introduced as $\mathcal{H} = \hbar \mathbf{\Omega_k} \cdot \mathbf{S}$ with $\mathbf{S}$ being the charge carrier spin operator, τ is the scattering time, and the angular brackets denote the averaging over the thermal distribution. The estimates show that τ_s can range in picoseconds to nanoseconds scale for a realistic set of system parameters. Also, fluctuations of the spin-orbit interaction caused, e.g., by random electric fields (as well as the Elliott-Yafet mechanism) can result in the spin relaxation of free charge carriers [19, 20].
In semiconductors under conditions of optical orientation, where spin-polarized carriers are photogenerated, the Bir-Aronov-Pikus mechanism can also be important [21]. This mechanism is related to the electron-hole exchange interaction described by the Hamiltonian

$$\mathcal{H} = \pi a_B^3 \hat{D} \delta(\mathbf{r}_{\rm e} - \mathbf{r}_{\rm h}) \delta_{\mathbf{k}_e + \mathbf{k}_h, \mathbf{k}'_e + \mathbf{k}'_h}. \tag{S4}$$

Here $\hat{D}$ is the operator acting on the electron and hole spins and describes the exchange interaction, a_B is the exciton Bohr radius, $\mathbf{r}_{\rm e}$ ($\mathbf{r}_{\rm h}$) is the electron (hole) position-vector, $\mathbf{k}_{\rm e}$ ($\mathbf{k}_{\rm h}$) is the electron (hole) wavevector and primes denote the wavevectors after the electron-hole collision. The operator $\hat{D} = \hat{D}_{\rm short} + \hat{D}_{\rm long}$ contains both the short-range and long-range contributions:

$$\hat{D}_{\rm short} = \Delta(\mathbf{S}_{\rm e} \cdot \mathbf{S}_{\rm h}), \quad \hat{D}_{\rm long} = \hbar\omega_{LT} \left[1 - \left(\hat{\mathbf{J}} \cdot \frac{\mathbf{K}}{K} \right)^2 \right]. \tag{S5}$$

Here Δ is the short-range interaction parameter, $\hbar\omega_{LT}$ is the longitudinal-transverse splitting of the bulk exciton, $\mathbf{K} = \mathbf{k}_{\rm e} + \mathbf{k}_{\rm h}$ is the exciton translational wavevector, and $\hat{\mathbf{J}}$ is the matrix of angular momentum J describing the states of the bright exciton triplet. For free carriers the Bir-Aronov-Pikus mechanism is efficient if there is residual doping or one type of carriers is efficiently depolarized by other means: In both cases the majority of collisions takes place between polarized electrons and unpolarized holes (or vice versa) and the spin relaxation rate can be roughly estimated as

$$\frac{1}{\tau_s^{(BAP)}} \sim \frac{\langle \hat{D}^2 \rangle}{E_B^2} \tau_{eh}^{-1}, \tag{S6}$$

with $\langle \hat{D}^2 \rangle$ being the appropriately averaged spin-flip matrix element of the operator $\hat{D}$ in Eq. (S5), τ_{eh} is the effective electron-hole scattering time and $E_B = \hbar^2/(2m_e a_B)^2$ is the Bohr energy.
Importantly, the exchange interaction results also in the depolarization of bound electron-hole pairs [22]. In this case the spin dephasing rate is associated with the characteristic value of the exciton fine structure splitting $\delta/\hbar$ resulting from Eq. (S4) [23].
For localized carriers an important contribution to the spin dephasing is provided by the hyperfine interaction with the nuclear spins (discussed below) [24] and also, in the presence of a magnetic field, by the carrier g-factor fluctuations. The latter effect contributes to the carrier spin dephasing rate in the ensemble with the rate

$$\frac{1}{T_2^*} \sim \frac{\Delta g \mu_B |B|}{\hbar}, \tag{S7}$$

providing the characteristic $T_2^* \propto 1/B$ dependence observed in experiment (Figure 2e of the main text) [25], Δg in Eq. (S7) is the g-factor spread and μ_B is the Bohr magneton.

Table S1: Major abundant non-zero nuclear spin isotopes in $FA_{0.9}Cs_{0.1}PbI_{2.8}Br_{0.2}$. The table columns give: isotope number, natural abundance α, nuclear spin I, relative dipole moment of the isotope μ relative to the nuclear magneton μ_N, gyromagnetic ratio γ.

isotope	α	I	μ/μ_N	γ [MHz T^{-1}]
^{207}Pb	22.1%	1/2	0.58	8.882
^{127}I	100%	5/2	2.81	8.578
^{79}Br	50.7%	3/2	2.1	10.704
^{81}Br	49.3%	3/2	2.27	11.538
^{133}Cs	100%	7/2	2.58	5.623
^{1}H	100%	1/2	2.8	42.577
^{14}N	100%	1	0.4	3.077
^{13}C	1%	1/2	0.7	10.708

S2.3 Hyperfine interaction

We turn now to the hyperfine interaction of the carrier spins with the host lattice nuclei. This interaction is the driving force of the spin dynamics of localized carriers. The hyperfine interaction both for the electron, $\mathbf{S}_e$, and hole, $\mathbf{S}_h$, spins with the spin of a single nucleus, $\mathbf{I}$, in bulk perovskite crystals can be written in the form [25, 24]

$$\mathcal{H}_{\mathrm{hf,e(h)}} = A_{\mathrm{e(h)}} v_0 |\varphi_{\mathrm{e(h)}}(\mathbf{R})|^2 (\mathbf{I}\cdot\mathbf{S}_{\mathrm{e(h)}}). \tag{S8}$$

Here $\mathbf{R}$ is the position of the nucleus, $\varphi_e(\mathbf{R})$ ($\varphi_h(\mathbf{R})$) is the electron (hole) envelope function, v_0 is the unit cell volume, and A_e (A_h) are the corresponding hyperfine constants. The scalar-product form of the hyperfine interaction in Eq. (S8) follows from the symmetry of the system: The valence band and conduction band states transform according to the irreducible representations R_6^+ and R_6^- of the O_h point symmetry group, resulting in the direct product $R_6^\pm \times R_6^\pm = \Gamma_1^+ + \Gamma_4^+$. Thus, the interaction with the effective magnetic field produced by the nucleus $\propto \mathbf{I}$ is isotropic. It can be expected that the symmetry reduction will produce a small anisotropy of the hyperfine interaction that is neglected in what follows. Preliminary model considerations and estimates of the hyperfine interaction in perovskite crystals were performed in Ref. [25]. The situation with the hyperfine coupling in the valence band is straightforward: The valence band Bloch wavefunctions have a considerable contribution of the s-type lead orbitals, which dominate the hyperfine coupling with the ^{207}Pb isotope (abundance $\alpha = 22.1\%$) with the hyperfine coupling constant $A_h(^{207}\mathrm{Pb}) \approx 100|C_s^{v.b.}|^2$ μeV [25], where the coefficient $C_s^{v.b.}$ is introduced in Eq. (S1a). The coefficient $C_s^{v.b.}$ is significant (and can be in the range $\sim 1/3\ldots 1$, depending on the microscopic model of the band structure which makes it possible to neglect the contribution of the p-orbitals of the halogens to the valence band hyperfine interaction, thus the hyperfine coupling constant in the valence band is roughly several 10's to 100 μeV depending on $C_s^{v.b.}$. The situation is much less obvious for the conduction band electrons. According to Refs. [10, 26], the conduction band is mainly formed by the p-orbitals of lead with an admixture of the s-orbitals of halogen. The estimates according to Refs. [27, 28] show that the hyperfine coupling constant with ^{207}Pb for the conduction electrons $A_e(^{207}\mathrm{Pb}) \approx 6|C_p^{c.b.}|^2$ μeV. For the halogen the calculations show that $A_e(^{127}\mathrm{I}) \approx 85|C_s^{c.b.}|^2$ μeV and $A_e(^{79}\mathrm{Br}) \approx A_e(^{81}\mathrm{Br}) \approx 90|C_s^{c.b.}|^2$ μeV, where $C_s^{c.b.}$ is the admixture coefficient of the s-shell orbitals of halogen to the mainly p-shell conduction band function, see Eq. (S1b). For a not too large admixture $|C_s^{c.b.}|^2 \sim 5\%\ldots 10\%$, we obtain conduction band hyperfine coupling constants on the order of several μeV. Note that both for the valence and conduction band the hyperfine coupling constants are positive because all isotopes have positive magnetic moments. We correct here the inconsistency in the discussion of the conduction band hyperfine coupling in Ref. [25] (where the contribution due to the p-orbitals of halogen has been evaluated). Note that the order of magnitude of the hyperfine interaction in the conduction band of several μeV is the same for the Pb, I and Br isotopes. The parameters of the abundant non-zero nuclear spin isotopes are given in Table. S1.

S2.4 Dynamic nuclear polarization

The scalar-product form of the hyperfine coupling Hamiltonian (S8) makes it possible to apply the standard theory of dynamic nuclear polarization (DNP) [29, 13, 24, 25]. In what follows we assume that the carriers are localized, otherwise the dynamic nuclear polarization is quite inefficient. The flip-flop processes result in the transfer of angular momentum from the carriers to the nuclei and the build-up of dynamic nuclear polarization, see the scheme in Figure 3**a** of the main text.

$$\langle \mathbf{I} \rangle = \ell \frac{4I(I+1)}{3} \frac{\mathbf{B}(\mathbf{B} \cdot \langle \mathbf{S} \rangle)}{B^2}. \tag{S9}$$

Here $\langle \mathbf{S} \rangle = \langle \mathbf{S}_{\mathrm{e(h)}} \rangle$ is the steady-state polarization of the electron ($\langle \mathbf{S}_{\mathrm{e}} \rangle$) or hole ($\langle \mathbf{S}_{\mathrm{h}} \rangle$) induced by optical orientation[†], $0 < \ell < 1$ is the leakage factor describing the relative efficiency of the hyperfine coupling-unrelated nuclear spin flip processes. For the general discussion we omit further in this section the subscripts e or h denoting the type of the carrier. The nuclear polarization builds up along the direction of the projection of the photocreated spin onto the magnetic field. The dynamic nuclear polarization $\langle \mathbf{I} \rangle$ produces the Overhauser field acting on the carrier spins

$$\mathbf{B}_{\mathrm{N}} = \frac{\alpha A \langle \mathbf{I} \rangle}{g \mu_B}, \tag{S10}$$

where α is the abundance of the corresponding isotope. If several isotopes are relevant, the total Overhauser field results from summation of the fields of the individual isotopes. The presence of the Overhauser field gives rise to a change of the carrier spin precession frequency: In the presence of DNP the Larmor frequency reads

$$\boldsymbol{\omega}_{\mathrm{L}} = \frac{g \mu_B}{\hbar} (\mathbf{B} + \mathbf{B}_{\mathrm{N}}) = \frac{g \mu_B \mathbf{B}}{\hbar} + \frac{\alpha A}{\hbar} \langle \mathbf{I} \rangle. \tag{S11}$$

Depending on the direction of the DNP provided by $\langle \mathbf{I} \rangle$ and the g-factor sign, the Larmor precession frequency can increase or decrease in the presence of DNP, see Figure 3 in the main text.
The efficiency of the DNP is determined by the leakage factor [24]

$$\ell = \frac{T_{1\mathrm{N}}}{T_1} = \frac{T_d}{T_1 + T_d}, \tag{S12}$$

in Eq. (S9), which describes the ratio of the total longitudinal nuclear spin relaxation time, $T_{1\mathrm{N}}^{-1} = T_1^{-1} + T_d^{-1}$, to the hyperfine-coupling-induced spin-flip time T_1, the time T_d describes the nuclear spin-flips due to the processes unrelated to the hyperfine coupling. The hyperfine coupling induced nuclear spin-flip rate can be estimated within the correlation time approximation for the carrier spin dynamics as

$$\frac{1}{T_1} \sim \left(\frac{A}{\hbar N_c} \right)^2 \frac{F \tau_c}{1 + (\omega_{\mathrm{L}} \tau_c)^2}. \tag{S13}$$

Here N_c is the number of unit cells within the carrier orbit[*], F is the probability of finding the carrier at the localization site, i.e., the average occupancy of the site, τ_c is the carrier correlation time and ω_{L} is given by Eq. (S11). In this equation we, as above, neglected the nuclear Zeeman splitting as it is small compared to that of the carriers. The denominator $1 + (\omega_{\mathrm{L}} \tau_c)^2$ describes the suppression of the nuclear spin flip processes because of the mismatch of the electron or hole and nuclear Zeeman energies. Naturally, a larger hyperfine coupling, generally, results in a larger nuclear spin flip rate T_1^{-1}, higher ℓ and, accordingly, higher DNP.
Importantly, depending on the directions of $\mathbf{B}$ and $\langle \mathbf{I} \rangle$ and the signs of A and g, the DNP can result either in an increase or a decrease in ω_L and, accordingly, in a decrease or an increase of T_1^{-1}. Indeed,

[†]The thermal polarization of carriers and nuclei can be neglected in our case. We also disregard the Knight field effect on the nuclei and assume that the external magnetic field exceeds by far the local fields acting on the nuclear spins via the dipole-dipole interactions.

[*]For simplicity we employ the box model assuming homogeneous hyperfine interaction within the charge carrier localization volume, see also Eq. (S15) below.

for parallel $\alpha A\mathbf{I}$ and $g\mu_B\mathbf{B}$ the Larmor precession frequency increases yielding an increase in the mismatch of the Zeeman splittings and suppressing T_1^{-1} and DNP. If $\alpha A\mathbf{I}$ and $g\mu_B\mathbf{B}$ are antiparallel, the DNP results in a reduction of the charge carrier Zeeman splitting (decrease of ω_L) and an increase of T_1^{-1}. Note, that in conventional semiconductor nanostructures this may give rise to a bistable behavior of the DNP [24].

Variation of the temperature or pump power can result in a variation of τ_c and F, yielding a different dependence of the Overhauser field on these two parameters. For instance, in the limit of short correlation times $\omega_\mathrm{L}\tau_c \ll 1$ the nuclear spin-flip rate $T_1^{-1} \propto F\tau_c$ and a temperature increase can result in the reduction of τ_c and F, e.g., due to the thermal delocalization of the charge carriers. Hence, a smaller T_1^{-1} and a smaller DNP are expected with increasing temperature and pump power. By contrast, for long correlation times, $\omega_\mathrm{L}\tau_c \gg 1$, $T_1^{-1} \propto F/\tau_c$ and the DNP temperature dependence can be non-trivial. Also, with increasing pump power, not only $\langle\mathbf{S}\rangle$ in Eq. (S9) increases, but also τ_c can be reduced yielding a saturation in DNP.

Let us now address in more detail the differences of the DNP induced by electrons and holes. Interestingly, the experiment demonstrates an Overhauser field acting on the electron spin, Figure 3e of the main text, and accordingly the build-up of DNP, despite the smaller hyperfine interaction constants in the conduction band. On the one hand, it could be assumed that the lead spins polarized via the hyperfine interaction with holes produce the Overhauser field acting on the electron spins. However, it is commonly accepted that the electrons and holes are spatially separated in the studied perovskite samples, as also confirmed by the TRKR experiments [25]. Also, this scenario can be ruled out by comparing the nuclei-induced shifts of the electron and hole spin precession frequencies as functions of the pump power, compare Figures 3f and 3e in the main text with respect of the temperature effect. While for the Overhauser field acting on the hole spins a saturation and even a non-monotonous behavior are observed depending on the temperature, the Overhauser field acting on the electron is a linear function of the pump power and is almost unaffected by the temperature. Thus, the very same nuclei cannot produce the fields acting on the electron and hole spins in the studies sample.

Thus, the electron polarizes the nuclear spins of the lead (unaffected by the hyperfine interaction with the holes) or of the halogen. Their similar values of the hyperfine interaction make it difficult to distinguish the isotopes from the viewpoint of the theory. However, the ODNMR experiments show that there is almost no effect of the RF field on the Overhauser field experienced by the electrons at the ^{207}Pb resonance frequency. Thus, it is likely that the DNP induced and experienced by the electrons comes from the halogen isotopes, Br and I in our case. Note that the large spin of iodine ($I = 5/2$) makes these nuclei particularly sensitive to quadrupole splittings which can, in our samples, be caused by inhomogeneous strain and electric field gradients, making it difficult to observe the ODNMR of these nuclei.

Note that for photoexcitation of electron-hole pairs in perovskites by circularly polarized light, the spins of electron and hole are parallel to each other, thus the direction of the dynamical nuclear polarization is the same regardless of the type of carrier. At the same time, the direction of the Overhauser field is controlled also by the sign of the carrier g-factor, Eq. (S11), which gives rise to a difference in the Overhauser field signs as schematically shown in Figure 3a and confirmed experimentally in Figures 3e,f of the main text.

So far, the DNP has been considered under the assumption that the photocreated carriers are separated after optical excitations and contribute to the DNP independently. In fact, this is not the only possible scenario. For instance, the spin coherence of the resident carriers and subsequent DNP can arise, like in conventional semiconductor quantum dot structures [30, 24], due to the resonant excitation of trions or $\mathrm{D}^0\mathrm{X}$ complexes with two identical charge carriers forming a spin singlet and an unpaired opposite charge carrier. Here, in principle, the DNP can be generated via the flip-flop of the unpaired carrier spin and the nuclear spin resulting in a non-trivial situation where the Overhauser field acting on the resident electron is created by the photocreated opposite charge carrier. For instance, in n-doped perovskites (or regions of the sample with resident electrons) the DNP acting on the electrons would be caused by the photo-holes then. However, this situation does not seem to be the case in the experiment: Indeed, on the basis of the theoretical analysis the hole mainly interacts with the Pb isotopes, and the ODNMR of the

lead is indeed observed in the Overhauser field acting on the holes rather than on the electrons.

S2.5 Nuclear spin fluctuations

We estimate the dispersion of the carrier Larmor frequency due to the hyperfine coupling with the host lattice nuclei. Following Ref. [24] we recast $\langle \omega_{\rm L}^2 \rangle = (g\mu_B/\hbar)^2 \langle B_{\rm N}^2 \rangle$ as

$$\langle \omega_{\rm L}^2 \rangle = \frac{1}{N_c} \sum_j \frac{I_j(I_j+1)}{\hbar^2} \alpha_j \left(A_j\right)^2 , \tag{S14}$$

where N_c is the number of the unit cells within the charge carrier localization volume,

$$N_c = v_0 \int |\varphi(\mathbf{r})|^4 d\mathbf{r}, \tag{S15}$$

with v_0 being the unit cell volume, and the summation is carried out over the relevant nuclei in the unit cell enumerated by j. The nuclear fluctuations provide the spin dephasing of localized electrons and holes at zero or small magnetic fields. The characteristic spin dephasing time

$$T_{2,0}^* \sim \frac{1}{\sqrt{\langle \omega_{\rm L}^2 \rangle}}. \tag{S16}$$

We make a rough estimate using the hyperfine coupling constants discussed in Sec. S2.3. For the hole spin dephasing related to the hyperfine interaction with ^{207}Pb ($I = 1/2$, $\alpha = 22.1\%$) we take $|C_s^{v.b.}|^2 = 1/3$ and get $A_{\rm h} \approx 33$ μeV. For the $N_{c,{\rm h}} = 10^4$, we have $\sqrt{\langle \omega_{\rm L,h}^2 \rangle} \approx 0.2$ ns^{-1} and $T_{2,0}^* \approx 5$ ns, in reasonable agreement with the experiment.† For the electron spin dephasing we assume that ^{127}I is relevant, take $|C_s^{c.b.}|^2 = 0.05$ and arrive at $A_{\rm e} = 4.25$ μeV. Taking into account that there are three iodine atoms within the unit cell we obtain the electron $T_{2,0}^* \approx 8$ ns for $N_{c,{\rm e}} = 7 \times 10^4$, i.e., for a somewhat larger electron localization volume as compared with that of the hole. The corresponding localization lengths of the carriers (evaluated taking into account the lattice constant of about 0.6 nm) are about 10 nm, see also [25]. We stress that these estimates can be considered as a general guide only, further microscopic calculations are needed to firmly establish the hyperfine coupling parameters in perovskites.
The estimate of the $N_{c,{\rm h}}$ obtained above and the experimental value of the Knight field $B_{\rm K} \approx 2$ mT obtained from ODNMR allows us to estimate the spin polarization degree of holes achieved in our experiment:

$$P_{\rm h} = 2\langle S_{\rm h} \rangle = 2 \frac{B_{\rm K}}{g_{\rm h}\mu_N} \frac{N_{c,{\rm h}}}{A_{\rm h}} \approx 6.6\%, \tag{S17}$$

for the previously quoted values of $A_{\rm h} \approx 33$ μeV and $N_{c,{\rm h}} = 10^4$.

S3 Experimental results

S3.1 Time-resolved Kerr rotation signals with applied RF

Time-resolved Kerr rotation signals with (red line) and without (black) applied RF of 6.66 MHz are shown in Figure S2a. They are measured in a magnetic field of 750 mT, $\varphi = 60°$. The used RF corresponds to the NMR resonance detected via the maximal changes in the KR amplitude measured at 320 ps time delay, see Figure S2b. The applied RF reduces the DNP, i.e., reduces the Overhauser field by $\Delta B_{\rm N} = B_{\rm N}(\text{no RF}) - B_{\rm N}(\text{with RF})$. For instance, as one can see in Figure S2a, with applied RF the Larmor precession period of the holes (the signal component with larger amplitude and smaller Larmor frequency) becomes shorter. This is most clearly seen at time delays exceeding 600 ps. Fits of the KR signals allow us to decompose them into the hole and electron components, which are shown individually in Figures S2c,d. For the holes (Figure S2c) $\Delta B_{\rm N,h} = 14.3$ mT, which corresponds to 40% of the Overhauser

†Note that in Ref. [25] we used $|C_s^{v.b.}|^2 = 1$ and obtained a shorter $T_{2,0}^* \approx 2$ ns for a similar localization volume.

field $B_{\mathrm{N,h}} = -37.3$ mT acting on the holes for these experimental conditions. For the electrons the RF effect is much smaller, but can be distinguished by an accurate fit as $\Delta B_{\mathrm{N,e}} = 0.18$ mT. It corresponds to a 5% reduction of $B_{\mathrm{N,e}} = 3.8$ mT.

As the RF coil covers an area of ≈ 1 mm^2 it depolarizes all lead spins independently of the origin of their polarization, via electrons or via holes. As the effect of RF on the hole and electron spin dynamics differs strongly, and in particular the electron-induced and -experienced DNP is almost unaffected by the RF excitation, it is likely that the Overhauser field for the electrons originates from different isotopes than for the holes, like the halide, as corroborated by our theoretical analysis, sec. S2.3.

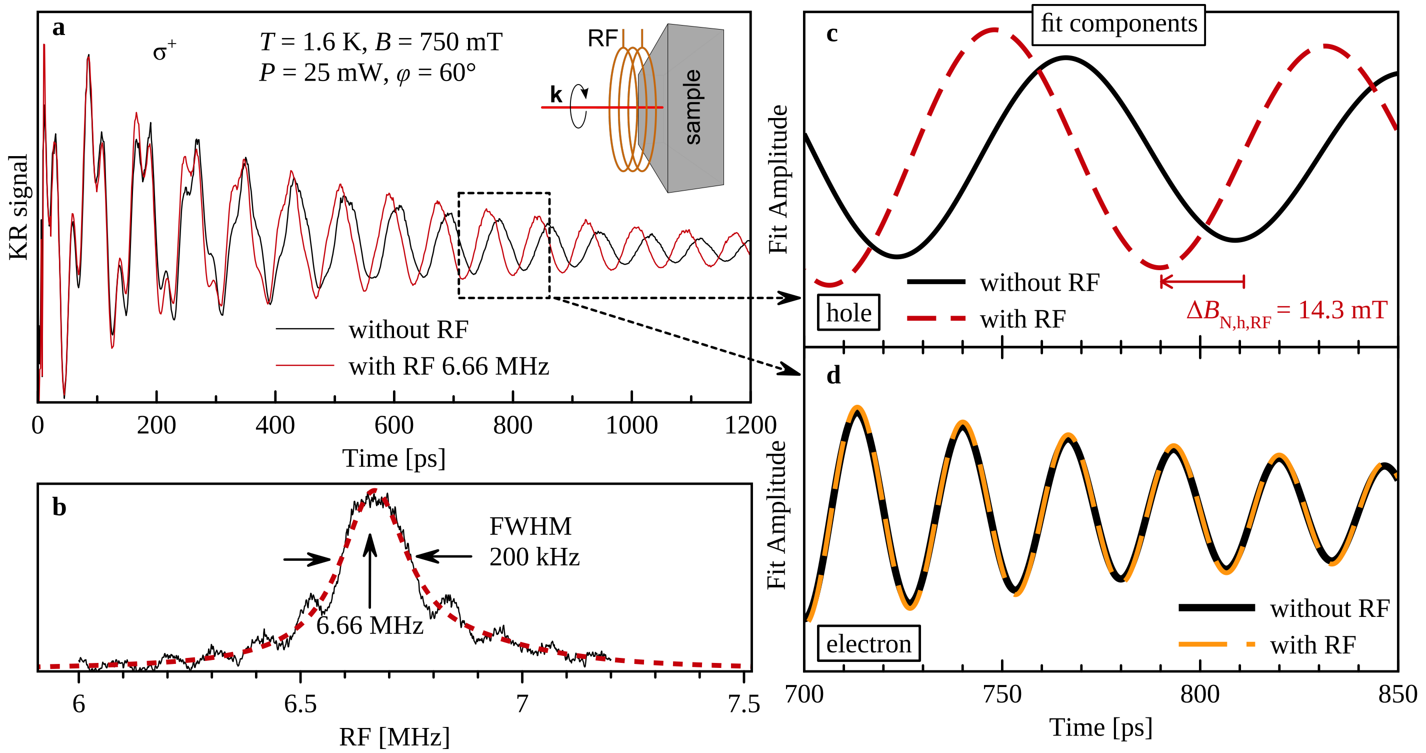

Figure S2: **a**, Example of time-resolved KR signals with (red) and without (black) resonant RF radiation at 6.66 MHz for $B = 750$ mT and $P = 25$ mW of σ^+ polarized pump. **b**, ODNMR resonance at given field, 200 kHz FWHM. **c,d**, Fit components after decomposition for hole (**c**) and electron **(d)** at long time delays. For the hole (dashed red and black lines) the difference is evident, while for the electron (solid black and dashed yellow line) no change is visible.

S3.2 Temperature dependence

Experimental data of the temperature dependence of the KR signal are given in Figure S3. The data are shifted vertically for clarity and not normalized. To keep the influence of laser-induced heating of charge carriers and lattice low, the pump power is set to 0.5 mW. Starting from $T = 6$ K a rapid decrease in both signal amplitude and spin dephasing time (T_2^*) is seen, resulting in a vanishing KR signal for temperatures exceeding 33 K. The decrease of T_2^* is presented in Figure 2 of the main text.

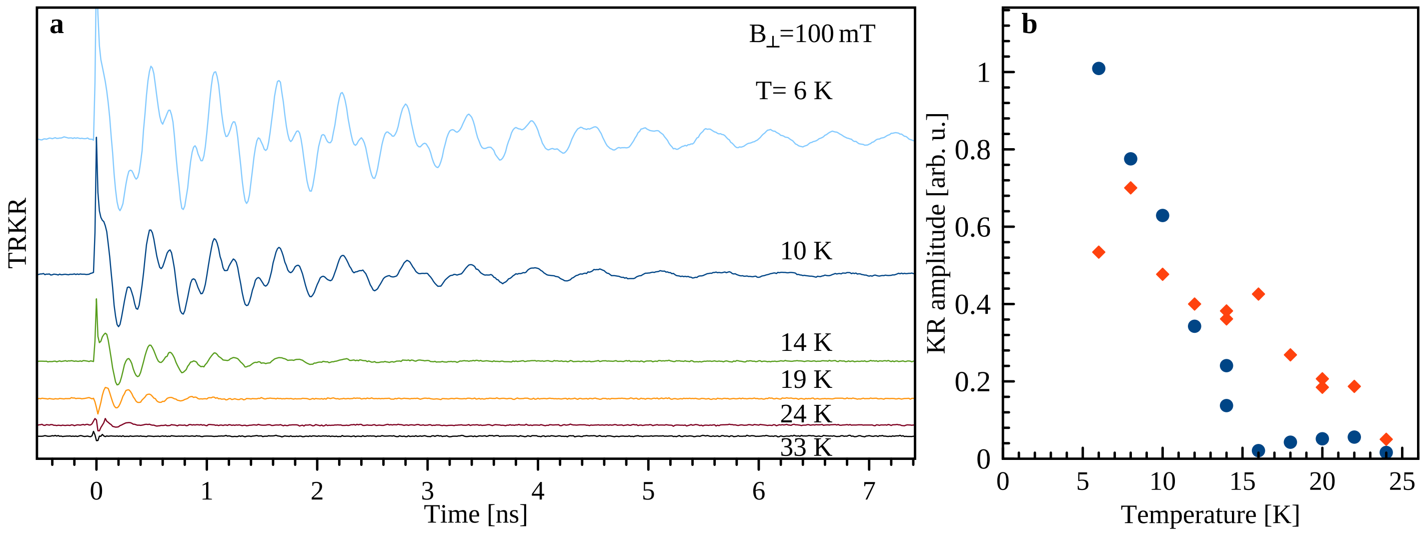

Figure S3: **a**, TRKR dynamics for various temperatures at $B_\perp = 100$ mT. Data are shifted vertically for clarity and not normalized. Pump power is kept low at 0.5 mW. **b**, KR amplitude at zero time delay versus temperature for electron (red) and hole (blue). The KR amplitude is interpolated via a fit and not phase corrected.

S3.3 ODNMR anisotropy

In general, the hyperfine interaction is anisotropic [31]. For instance, in Refs. [32, 33] the chemical shift or shielding of nuclei is demonstrated to be anisotropic. With the use of a 3D vector magnet, we are able to test the anisotropy by solely rotating the magnetic field and keeping all other experimental conditions constant. The magnetic field is rotated in the horizontal plane from Voigt ($\varphi = 90°$, $\mathbf{B} \perp \mathbf{k}$) to Faraday ($\varphi = 0°$, $\mathbf{B} \parallel \mathbf{k}$) field direction. A fixed time delay of 613 ps is set which is nominally half the period of the Larmor precession of the hole at $\varphi = 60°$, but is not rechecked for different angles in order to optimize the signal amplitude.

In Figure S4a ODNMR signals are shown for several angles. It is seen that for $\varphi = 60° \pm 20°$ pronounced ODNMR signals are obtained. The amplitude shows a maximum for $\varphi = 60°$ and decreases with tilting away from this orientation in both direction. This is shown in Figure S4b, where the normalized amplitude on its value at $\varphi = 60°$ is plotted. For ODNMR, a compromise between magnetic field tilting towards Faraday (providing larger DNP) and Voigt (better read-out via Kerr rotation) needs to be found [34]. The ODNMR resonance frequency is not changing significantly with angle Figure S4c, at least within the FWHM of about 170 kHz. Note that the line width here is broadened in comparison to the ODNMR shown in the main text due to a higher applied RF power by using an additional self-built audio amplifier not rated in the power output.

For comparison, from data of NMR spectroscopy it is useful to express the shift in units of parts per million, i.e. as relative shift to central position. The shift amounts up to 5000 ppm. This would be a large shift for high field NMR experiments (typical range $100 - 1000$ ppm) but is rather normal for low field ODNMR measurements where the denominator is small, i.e. the within sensitivity of our experiments. For illustration in Figure S4c, we specified an error given by the magnetic field accuracy of 0.5 mT (0.2%) immanent as reproducibility error for superconducting magnets, like remanence etc. Within the TRKR accuracy the ODNMR signal is nearly isotropic.

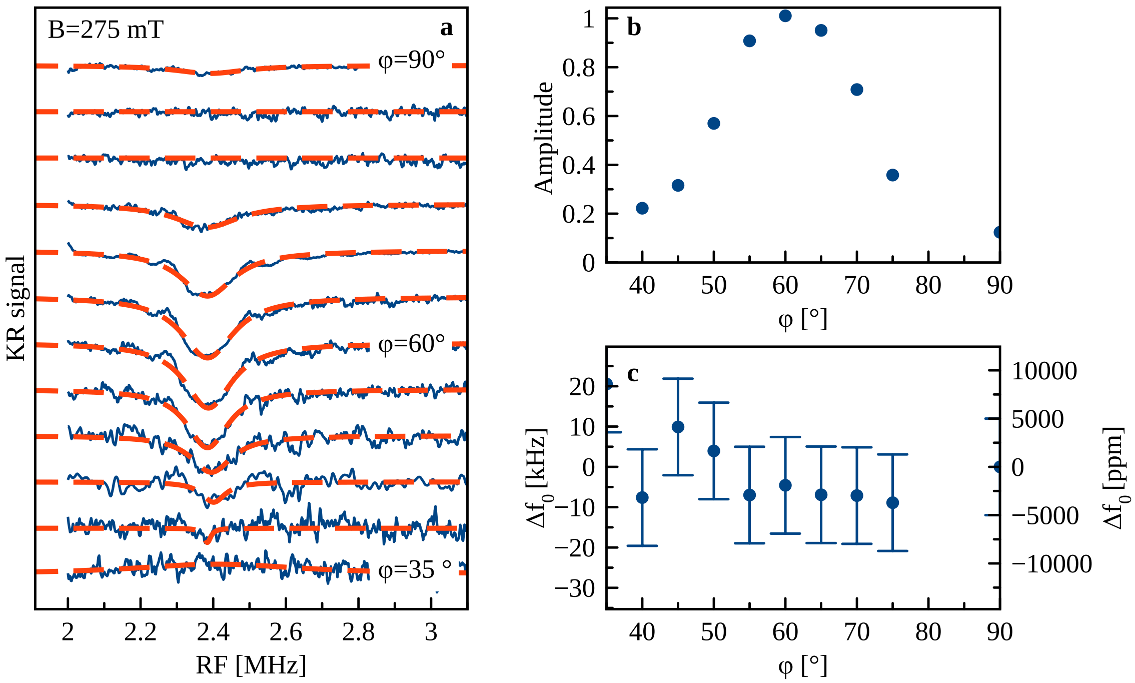

Figure S4: **a**, ODNMR signals (blue line) for different angles φ of the external magnetic field. $B = 275$ mT, $T = 1.6$ K, $P = 25$ mW, 613 ps time delay and σ^- pump polarization. The red dashed line shows a fit of the resonance with a Lorentz dip on top of an offset. Resonance FWHM is 170 kHz. **b**, Amplitudes of the ODNMR signal normalized on the maximum. **c**, Relative shift of the central ODNMR peak position $\Delta f_0 = f_\varphi - f_{0°}$, $f_0 = 2.39$ MHz.

S3.4 Carrier g-factor anisotropy

The g-factor anisotropy for electrons and holes is measured with the vector magnet, maintaining the optical alignment and rotating the magnetic field, Figure S5. The magnetic field orientation with respect to the incidence light vector $\mathbf{k}$ is sketched in Figure S5**d**, $\varphi = \theta = 0°$ which corresponds to $\mathbf{B}_{\parallel,z}$. Two planes, namely Voigt-Voigt ($\mathbf{B}_{\perp,x}$ to $\mathbf{B}_{\perp,y}$) and Faraday-Voigt ($\mathbf{B}_{\parallel}$ to $\mathbf{B}_{\perp,x}$) are measured. The g-factors for all other solid angles are assumed to be determinable by interpolation between these planes. The g-factor anisotropy is rather small in $FA_{0.9}Cs_{0.1}PbI_{2.8}Br_{0.2}$ crystals, not exceeding 3% for electrons and 4% for holes (details will be published elsewhere).

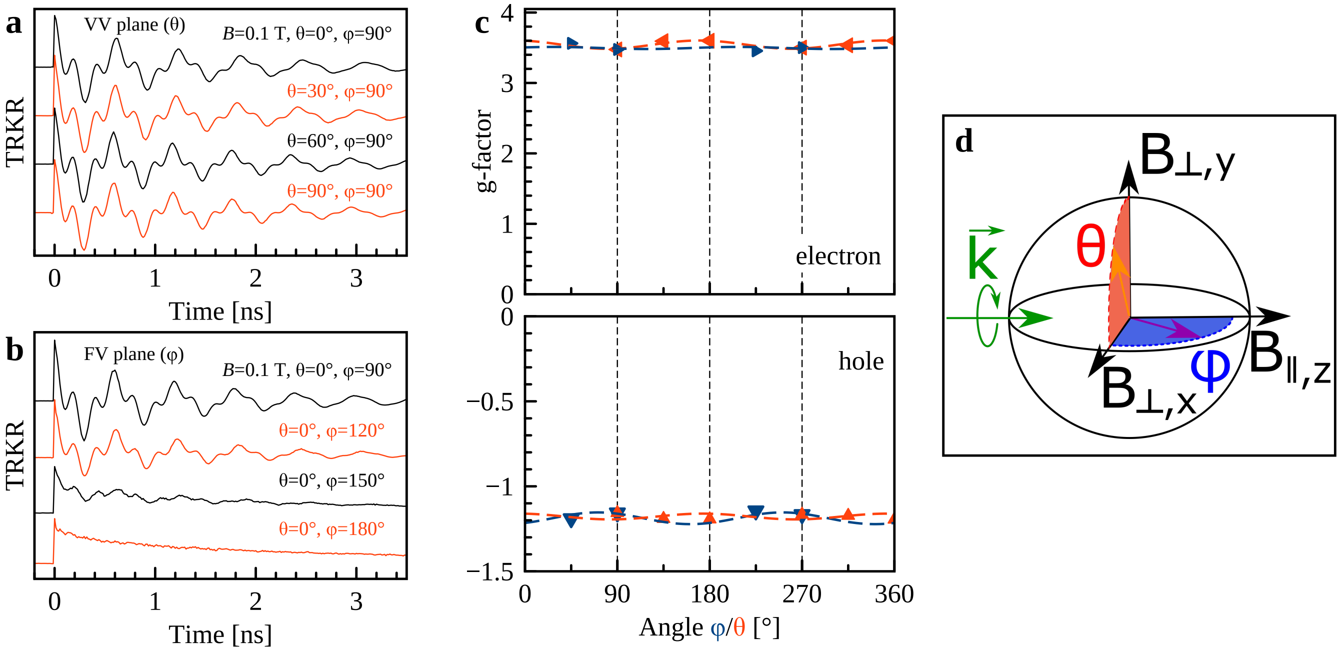

Figure S5: ***g*-factor anisotropy**. **a**, TRKR signals within the Voigt-Voigt plane ($\varphi = 90°$, θ scanned). **b**, TRKR signals within the Faraday-Voigt plane (φ scanned, $\theta = 0°$). $T = 1.6$ K, $P = 10$ mW. **c**, g-factor dependence on solid angle φ (blue), θ (red). **d**, Sketch of magnetic field orientation with respect to the incident light vector **k**. Red (blue) plane corresponds to Voigt-Voigt (Voigt-Faraday) plane. $\varphi = \theta = 0°$ corresponding to $\mathbf{B}_{\parallel,z}$.

S3.5 Time-resolved photoluminescence

We have measured recombination dynamics in the $FA_{0.9}Cs_{0.1}PbI_{2.8}Br_{0.2}$ crystal at a maximum of the photoluminescence (PL) line at a temperature of $T = 1.7$ K. PL was excited by a pulsed laser with a photon energy of 2.33 eV (wavelength of 532 nm) having a repetition frequency of 10 kHz and an average excitation power of 8 μW. The signal has been detected by an avalanche photodiode and a time-of-flight card with a time resolution of 30 ns. Recombination dynamics in a wide temporal range up to 100 μs is shown in Figure S6. One can see that the recombination dynamics is strongly non-exponential. It has recombination times ranging from 0.2 up to 44 microseconds, which by far exceed the typical lifetimes of excitons and is characteristic for the recombination of spatially separated electrons and holes having considerable dispersion in their separation lengths. This is in good agreement with our conclusions made on the spin-dependent experiments, that at low temperatures in this crystal we have a considerable concentration of spatially separated long-living electrons and holes (we call them resident electrons and holes in the main text).

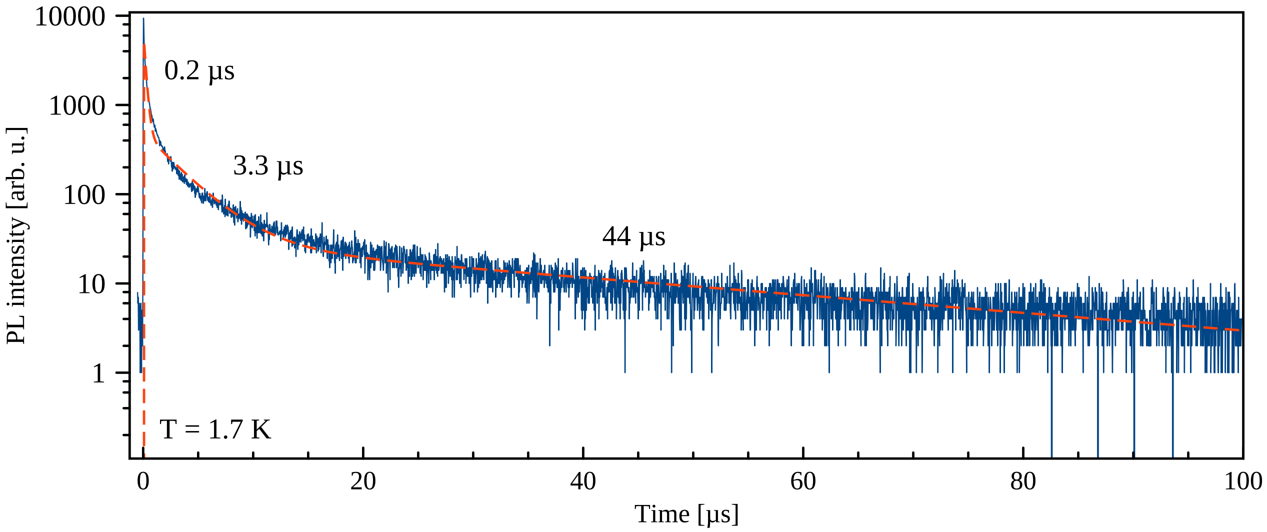

Figure S6: **Time-resolved photoluminescence of $FA_{0.9}Cs_{0.1}PbI_{2.8}Br_{0.2}$ crystal**. Recombination dynamics is measured at the maximum of PL line (at 1.495 eV), see blue spectrum in Figure 1b in the main text, at a temperature of $T = 1.7$ K. The dynamics is fitted with a three-exponential function having characteristic times of 0.2 μs, 3.3 μs and 44 μs. The corresponding fit is shown by a dashed red line.

References

[1] Walsh, A. WMD-group/hybrid-perovskites. URL `https://github.com/WMD-group/hybrid-perovskites`.

[2] Nazarenko, O., Yakunin, S., Morad, V., Cherniukh, I. & Kovalenko, M. V. Single crystals of caesium formamidinium lead halide perovskites: solution growth and gamma dosimetry. *NPG Asia Materials* **9**, e373 (2017).

[3] Zhumekenov, Ayan A. *et al.* Formamidinium lead halide perovskite crystals with unprecedented long carrier dynamics and diffusion length. *ACS Energy Lett.* **1**, 32–37 (2016).

[4] McMeekin, D. P. *et al.* A mixed-cation lead mixed-halide perovskite absorber for tandem solar cells. *Science* **351**, 151–155 (2016).

[5] Jeon, N. J. *et al.* Compositional engineering of perovskite materials for high-performance solar cells. *Nature* **517**, 476–480 (2015).

[6] Goldschmidt, V. M. Die Gesetze der Krystallochemie. *Naturwissenschaften* **14**, 447–485 (1926).

[7] Li, Z. *et al.* Stabilizing perovskite structures by tuning tolerance factor: Formation of formamidinium and cesium lead iodide solid-state alloys. *Chemistry of Materials* **28**, 284–292 (2016).

[8] Whitfield, P. S. *et al.* Structures, phase transitions and tricritical behavior of the hybrid perovskite methyl ammonium lead iodide. *Scientific Reports* **6**, 35685 (2016).

[9] Even, J. Pedestrian guide to symmetry properties of the reference cubic structure of 3D all-inorganic and hybrid perovskites. *J. Phys. Chem. Lett.* **6**, 2238–2242 (2015).

[10] Boyer-Richard, S. *et al.* Symmetry-based tight binding modeling of halide perovskite semiconductors. *J. Phys. Chem. Lett.* **7**, 3833–3840 (2016).

[11] Koster, G. F., Dimmock, J. D., Wheeler, R. G. & Statz, H. *The Properties of the Thirty-Two Point Groups* (MIT Press, Cambridge, MA, USA, 1963).

[12] Nestoklon, M. O. Tight-binding description of inorganic lead halide perovskites in cubic phase. *Com. Mat. Sci* **196**, 110535 (2021).

[13] Meier, F. & Zakharchenya, B. P. (eds.) *Optical Orientation* (Elsevier, Amsterdam, 1984).

[14] Dyakonov, M. I. (ed.) *Spin Physics in Semiconductors* (Springer International Publishing AG, 2017).

[15] Kim, M., Im, J., Freeman, A. J., Ihm, J. & Jin, H. Switchable $S = 1/2$ and $J = 1/2$ Rashba bands in ferroelectric halide perovskites. *PNAS* **111**, 6900–6904 (2014).

[16] Kepenekian, M. *et al.* Rashba and Dresselhaus effects in hybrid organic-inorganic perovskites: from basics to devices. *ACS Nano* **9**, 11557–11567 (2015).

[17] Niesner, D. *et al.* Giant Rashba splitting in $CH_3NH_3PbBr_3$ organic-inorganic perovskite. *Phys. Rev. Lett.* **117**, 126401 (2016).

[18] Dyakonov, M. I. & Perel', V. I. Spin relaxation of conduction electrons in noncentrosymmetric semiconductors. *Sov. Phys. Solid State* **13**, 3023–3026 (1972).

[19] Gryncharova, E. I. & Perel', V. I. Relaxation of nuclear spins interacting with holes in semiconductors. *Sov. Phys. Semicond.* **11**, 997 (1977).

[20] Glazov, M. M., Sherman, E. Y. & Dugaev, V. K. Two-dimensional electron gas with spin–orbit coupling disorder. *Physica E* **42**, 2157–2177 (2010).

[21] Bir, G. L., Aronov, A. G. & Pikus, G. E. Spin relaxation of electrons due to scattering by holes. *Sov. Phys. JETP* **42**, 705 (1975).

[22] Maialle, M. Z. Spin dynamics of localized excitons in semiconductor quantum wells in an applied magnetic field. *Phys. Rev. B* **61**, 10877–10882 (2000).

[23] Ivchenko, E. L. *Optical Spectroscopy of Semiconductor Nanostructures* (Alpha Science, Harrow UK, 2005).

[24] Glazov, M. M. *Electron & Nuclear Spin Dynamics in Semiconductor Nanostructures* (Oxford University Press, Oxford, 2018).

[25] Belykh, V. V. *et al.* Coherent spin dynamics of electrons and holes in $CsPbBr_3$ perovskite crystals. *Nature Communications* **10**, 673 (2019).

[26] Targhi, F. F., Jalili, Y. S. & Kanjouri, F. $MAPbI_3$ and $FAPbI_3$ perovskites as solar cells: Case study on structural, electrical and optical properties. *Results in Physics* **10**, 616–627 (2018).

[27] Koh, A. & Miller, D. Hyperfine coupling constants and atomic parameters for electron paramagnetic resonance data. *Atomic Data and Nuclear Data Tables* **33**, 235–253 (1985).

[28] Morton, J. & Preston, K. Atomic parameters for paramagnetic resonance data. *J. Magn. Reson. (1969)* **30**, 577–582 (1978).

[29] Abragam, A. *The Principles of Nuclear Magnetism* (University Press, Oxford, 1994).

[30] Urbaszek, B. *et al.* Nuclear spin physics in quantum dots: An optical investigation. *Rev. Mod. Phys* **85**, 79–133 (2013).

[31] Weil, J. A. & Bolton, J. R. *Hyperfine (A) Anisotropy in Electron Paramagnetic Resonance* (Wiley, John & Sons Ltd., 2006) pp. 118–157.

[32] Sharma, S., Weiden, N. & Weiss, A. ^{207}Pb and ^{205}Tl NMR on perovskite type crystals $APbX_3$ (A = Cs, Tl, X = Br, I). *Zeitschrift für Naturforschung A* **42**, 1313–1320 (1987).

[33] Nolle, A. ^{207}Pb magnetic shielding anisotropy in $Pb(NO_3)_2$, $PbCO_3$, $PbCrO_4$, $PbMo0_4$ and $PbWO_4$ by Fourier transform NMR. *Zeitschrift für Naturforschung A* **32a**, 964–967 (1977).

[34] Kalevich, V. K. & Korenev, V. L. Optical polarization of nuclei and ODNMR in GaAs/AlGaAs quantum wells. *Appl. Magn. Reson* **2**, 397–411 (1991).

Table S2: Notations used in the paper.

Notation	Definition
$\mathbf{B}$ -	external magnetic field
$\mathbf{B}_{\mathrm{K,e}}$ -	Knight field of electron
$\mathbf{B}_{\mathrm{K,h}}$ -	Knight field of hole
$\mathbf{B}_{\mathrm{N,e}}$ -	Overhauser field on electron
$\mathbf{B}_{\mathrm{N,h}}$ -	Overhauser field on hole
g_{e} -	g factor of conduction band electron
g_{h} -	g factor of valence band hole
$\omega_{\mathrm{L,e}}$ -	Larmor precession frequency of electrons
$\omega_{\mathrm{L,h}}$ -	Larmor precession frequency of holes
ω_{L} -	Larmor precession frequency
T -	lattice temperature
$T_{1,\mathrm{N}}$ -	longitudinal spin relaxation time of nuclei
$T_{1,\mathrm{e}}$ -	longitudinal spin relaxation time of electron
$T_{1,\mathrm{h}}$ -	longitudinal spin relaxation time of hole
$T_{2,\mathrm{e}}^{*}$ -	spin dephasing time of electron ensemble
$T_{2,\mathrm{h}}^{*}$ -	spin dephasing time of hole ensemble
T_d -	nuclear spin-flip time unrelated to hyperfine coupling
$\mathbf{B}_{\perp}$ -	Voigt magnetic field (also $\mathbf{B}_{\perp,x}$ and $\mathbf{B}_{\perp,y}$)
$\mathbf{B}_{\parallel}$ -	Faraday magnetic field (also $\mathbf{B}_{\parallel,z}$)
$\lvert c.b.\rangle, \lvert v.b.\rangle$ -	conduction band, valance band wave function
$\lvert \mathcal{S}_n\rangle, (\lvert \mathcal{X}_n\rangle, \lvert \mathcal{Y}_n\rangle, \lvert \mathcal{Z}_n\rangle)$ -	s (p)-type wavefunction, subscript n denoting respective atom Pb, I
$C_s^{c.b.}, C_p^{c.b.}, C_s^{v.b.}, C_p^{v.b.}$ -	orbital admixture coefficients
$\frac{1}{\tau_s^{(DP)}}$ -	spin relaxation rate via Dyakonov-Perel' mechanism
Ω_k -	effective frequency of the carrier spin precession in spin-orbit field
τ -	scattering time
a_B -	exciton Bohr radius
$\hat{D}$ -	charge exchange interaction operator
$\mathbf{S}_{\mathrm{e(h)}}$ -	electron (hole) spin operator
$\mathbf{r}_{\mathrm{e(h)}}$ -	electron (hole) position-vector
$\mathbf{k}_{\mathrm{e(h)}}$ -	electron (hole) wavevector-vector, (primes denote scattering event)
$\hbar\omega_{LT}$ -	longitudinal-transverse splitting of bulk exciton
$\hat{J}$ -	matrix of angular momentum
$\mathbf{K}$ -	exciton translational wavevector
$\frac{1}{\tau_s^{(BAP)}}$ -	Bir-Aronov-Pikus relaxation rate
τ_{eh} -	electron-hole scattering time
$E_B = \hbar^2/2m_e a_B^2$ -	the Bohr energy
g -	g-factor
Δg -	spread of g-factor
$A_{\mathrm{e(h)}}$ -	electron (hole) hyperfine constant
v_0 -	unit cell volume
$\phi_{\mathrm{e(h)}}(\mathbf{R})$ -	electron (hole) envelope function
$\mathbf{I}$ -	nuclear spin
α	specific isotope abundance
l -	leakage factor
N_c -	unit cells within the charge carrier localization volume
F -	probability to find carrier at localization site
τ_c -	carrier correlation time
P_{h} -	polarization degree of holes
